# Seminal papers in urology: anticholinergic therapy vs. onabotulinumtoxina for urgency urinary incontinence

**DOI:** 10.1186/s12894-023-01273-y

**Published:** 2023-05-24

**Authors:** Nathan Shugg, Michael E. O’Callaghan

**Affiliations:** 1 Urology Unit, Flinders Medical Centre, Bedford Park, Australia; 2 College of Medicine and Public Health, Flinders University, Adelaide, Australia; 3 Discipline of Medicine, The University of Adelaide, Adelaide, Australia

## Abstract

In this critical review, we explore the study design, strengths, and limitations of landmark trial “Anticholinergic therapy vs. onabotulinumtoxinA for urgency urinary incontinence”. This trial was the first to directly compare two key treatment options for urge urinary incontinence – anticholinergic medication and intravesical botox, and still influences clinical guidelines a decade after publication. This non-inferiority, double-blinded, multi-centre randomised controlled trial administered Solifenacin or intra-detrusor botox to women, measuring outcomes six months post-treatment. Non-inferiority of the treatments was established, though Botox had a higher rate of retention and infection, with side effect profile rising as the key discriminator in selecting first-line therapy.

## The clinical problem

Urgency urinary incontinence (UUI) is a highly prevalent condition affecting up to 36.4% of individuals worldwide [1], with prevalence increasing with age. It is a common part of Overactive Bladder syndromes (OAB) [2].

Traditionally, anticholinergic medications have been utilised as mainstay agents in reducing incontinence episodes, with intravesical injection of OnabotulinumtoxinA (Botox) regarded as an option for resistant cases [3, 4]. This is still reflected in current guidelines, with both the American Urological Association (AUA) [5] and European Association of Urology (EAU) [6] non-neurogenic OAB Guidelines recommending behavioural modification as first-line therapy, followed by anticholinergic medications in patients who fail a conservative approach. Surgical management via intravesical Botox injection is therefore a third-line treatment, recommended specifically for UUI refractory to pharmacotherapy.

However, both therapy pathways carry side effects that can significantly impact quality of life (QoL) and treatment adherence, and though a meta-analysis in 2009 [8] found both options to be effective in isolation, it highlighted a dearth of evidence directly comparing these pathways.

### The landmark paper

“Anticholinergic Therapy vs. OnabotulinumtoxinA for Urgency Urinary Incontinence” [7], published November 2012 in The New England Journal of Medicine, sought to directly compare anticholinergic pharmacotherapy to intravesical Botox injection, evaluating efficacy in reducing urge incontinence episodes, as well as real-world QoL effects and adverse event profiles, with an aim to guide selection of initial treatment.

The study was a randomized, double-blind, double-placebo–controlled trial, which is commonly accepted as gold standard for investigating comparative efficacy of interventions. Two arms were defined, those initiated on anticholinergic medication (solifenacin 5 mg daily) who also underwent a single detrusor injection of saline, and those who received oral placebo and intravesical Botox (100 IU).

Treatment and follow-up continued for six months, with option for dose escalation in oral agent (first to solifenacin 10 mg daily, then trospium 60 mg daily) at 8-week intervals based on clinical response as defined by the Patient Global Symptom Control (PGSC) scale. In the Botox arm, placebo was escalated in the same pattern according to the same criteria. After six months, all oral medication was ceased, with a further six months of follow up to assess any continuing efficacy off-treatment.

Eligibility is clearly defined as women who had five episodes of urinary urge-predominant incontinence across a three-day bladder diary, and patients who had previously been on anticholinergic medication were not excluded though a two-week washout period was enforced. Mid-study change in anticholinergic agent or dose was not adjusted for, despite only a single dose of a single agent available for those in the Botox arm.

The study was powered to achieve 80% power (alpha 0.05) with recruitment of 121 participants in two arms. This calculation was based on a targeted endpoint of reduction in incontinence episodes across 6-months of 0.8 episodes per day with standard deviation of 2.1 episodes per day.

Data was collected from 10 different US sites, all urological or gynaecological centres, from a wide variety of states and demographic areas to promote generalisability, though all would be classified as metropolitan. Detailed in a protocol published prior to study initiation [8], randomisation used computer-assisted permuted blocks, stratified by previous exposure to anticholinergics and baseline number of UUI episodes. Patient demographics were consistent between study groups demonstrating successful randomisation, and mean participant characteristics of a female in the 5th decade of life with ~ 5x episodes of incontinence per day is typical of many patients treated for UUI.

Primary and secondary outcomes were well-defined, with a primary endpoint of change in incontinence episodes across 6-months as reported in monthly three-day bladder diaries. Secondary endpoints included scores on previously validated QoL questionnaires, and adverse events.

### Summary of outcomes

126 women were treated in the anticholinergic arm, with a reduction of 3.4 UUI episodes per day over six months. In the Botox arm, 121 women were treated, with a reduction of 3.3 UUI episodes per day (p-value = 0.81). Likewise, QoL score changes were comparable between arms, with reduction of symptom-severity scale score of 44.55 in the anticholinergic arm, and 44.08 in the Botox arm (p = 0.98). The combination of this data supports non-inferiority between the two management options.

Overall adverse event rate was similar between groups, with 69% of those on anticholinergics, and 73% of those who received intravesical Botox reporting at least one adverse event. However, differentiating the two arms was the exact nature of adverse event. No serious adverse events were attributable to study treatment.

Those in the anticholinergic group had statistically significant higher rates of dry mouth (46% vs. 31%, p = 0.02). Of note, dry mouth was not a significant trigger for drug withdrawal, indicating a low impact on QoL. While there was a trend towards higher rates of other typical anticholinergic side effects such as constipation and dry eyes, they did not reach statistical significance.

Conversely, the Botox group had significantly higher rates of urinary tract infection (UTI), with 33% vs. 13% (p < 0.001). They also had higher post-void residual volume (p < 0.001) with 9% of participants requiring intermittent self-catheterisation (ISC) at two weeks post-Botox. While this number reduced over the six-month study period, no women in the anticholinergic group required intermittent catheterisation.

Following cessation of treatment, those in the Botox arm had a trend towards greater long-term control off-treatment. However, this did not reach statistical significance.

Overall, both treatments were found to be effective to the same degree, with side effect profile being the primary delineator. Given the significant side effect of dry mouth in the anticholinergic group did not result in treatment cessation, it is likely that the higher rate of UTI and ISC represent the more significant burden to patient outcomes.

In practice, this study supports anticholinergic therapy remaining first-line for OAB management, primarily as risk-reduction for adverse events, with escalation of dose or change in agent considered if inadequate effect. Intravesical Botox should be considered second-line in treatment resistant cases, but with careful discussion of potential infection and retention, and guided by patient ability to identify these events and enact management such as self-catheterisation. This is reflected in current guidelines recommending Botox as a third-line agent, despite non-inferiority [6, 9].

### Assessment of evidence

“Anticholinergic Therapy vs. OnabotulinumtoxinA for Urgency Urinary Incontinence” [7] is a large, multicentre, randomised controlled trial which re-established the non-inferiority of these two key management options for urge incontinence, while highlighting the importance of adverse event profiles in treatment selection for this population.

Using the Cochrane risk-of-bias assessment tool (version 2) [10], we found this study to have a low risk of bias in all areas.

Although published 10 years ago, it still has ongoing significant impact on management discussions in current practice, with both AUA and EUA citing this study in respective OAB management guidelines.

Weaknesses of the study include the lack of sub-group analysis between participants who were dose-escalated or changed agent in comparison with the Botox arm. In addition, patients who previously had anticholinergic treatment were not excluded, and no sub-group analysis was performed to determine if prior treatment of any kind conveyed residual influence on efficacy outcomes. However, this was adjusted for within the randomisation process, indicating generalisability of outcomes regardless.

Overall, these factors do not detract from the outcomes and their ability to be translated to clinical decision making. Large sample-size randomised controlled trials of invasive surgical management such as intravesical injection are rare, and it is highly unlikely that another study will be safely or ethically conducted in the near term.

### Future research

Further targets for investigation could be intravesical Botox injection in anticholinergic-naïve populations specifically, and with escalating doses. Since this study, the clinical sphere has also been expanded by the introduction of beta-3 agonists such as Mirabegron, which have become alternative second-line pharmacological agents in current guidelines [5, 6]. These have the particular benefit of avoiding anticholinergic side effects, especially cognitive effects in an aging population [10], and have been proven particularly effective in men who have failed anticholinergic therapies [11], though not all countries can access this medication. Also promising are early studies establishing the non-inferiority of posterior tibial nerve stimulation[11].

As these new management options continue to be developed, direct comparative studies with newer agents may be necessary to demystify treatment selection for this complex yet prevalent condition once again.

## Data Availability

Not applicable.
